# Genetic variation in TBC1 domain family member 1 gene associates with the risk of lean NAFLD *via* high-density lipoprotein

**DOI:** 10.3389/fgene.2022.1026725

**Published:** 2023-01-12

**Authors:** Na Wu, Xiangyu Zhai, Fan Yuan, Jie Li, Dong Li, Jianying Wang, Lei Zhang, Yi Shi, Guang Ji, Guang He, Baocheng Liu

**Affiliations:** ^1^ Shanghai Innovation Center of Traditional Chinese Medicine Health Service, School of Public Health, Shanghai University of Traditional Chinese Medicine, Shanghai, China; ^2^ Bio-X Institutes, Key Laboratory for the Genetics of Developmental and Neuropsychiatric Disorders, Shanghai Jiao Tong University, Shanghai, China; ^3^ Graduate School of Sport Sciences, Waseda University, Saitama, Japan; ^4^ Zhangjiang Community Health Service Center of Pudong New District, Shanghai, China; ^5^ Institute of Digestive Diseases, Longhua Hospital, Shanghai University of Traditional Chinese Medicine, Shanghai, China

**Keywords:** lean NAFLD, TBC1D1 gene, high-density lipoprotein, mediation effect, polymorphisms

## Abstract

**Objective:** Non-alcoholic fatty liver disease (NAFLD) affects almost a quarter of the world’s population. Although NAFLD often co-exists with obesity, a substantial proportion of NAFLD patients are lean which is relatively unexplored. This study aimed to examine the association between genetic variation in candidate genes, e.g., *TBC1D1* and the risk of lean NAFLD in the elderly Chinese Han population.

**Methods:** This is an extension of the research on physical examination in the Zhanjiang community center including 5387 residents, Shanghai, China, in 2017. According to the classification in adult Asian populations, participants were categorized into four groups: lean NAFLD (BMI <23, *n* = 106), non-lean NAFLD (BMI ≥23, *n* = 644), lean non-NAFLD (BMI <23, *n* = 216) and non-lean non-NAFLD (BMI ≥23, *n* = 253).116 NAFLD-related candidate genes, which cover 179 single nucleotide polymorphisms (SNPs) were included in the KEGG enrichment analysis. Independent samples *t*-test was adopted for the group comparison. The associations between genetic variations with the specific phenotype in five genetic models were analyzed with the “SNPassoc” R package and rechecked with logistic regression analysis. Mediation models were conducted to explore whether the certain phenotype can mediate the association between SNPs and the risk of lean NAFLD.

**Results:** Compared with lean non-NAFLD individuals, lean NAFLD patients had higher BMI, low-density lipoprotein and triglyceride, and lower HDL. The AMPK signaling pathway, which includes *TBC1D1* and *ADIPOQ* genes, was the most significant (*p* < .001). The A allele frequency of rs2279028 in *TBC1D1* (*p* = .006) and G allele frequency of rs17366568 in *ADIPOQ* (*p* = .038) were significantly lower in lean NAFLD. The association between rs2279028 and the risk of lean NAFLD was mediated by HDL (*p* = .014). No significant mediation effect was found between rs17366568 and the risk of lean NAFLD.

**Conclusion:** This study, for the first time, indicated that rs2279028 of *TBC1D1* may contribute to the progression of lean NAFLD through HDL. This finding provides more evidence for exploring the mechanism of lean NAFLD and suggests practical solutions for the treatment of lean NAFLD.

## Introduction

As one of the public health problems, non-alcoholic fatty liver disease (NAFLD) affects almost a quarter of the world population and triggers the occurrence and deterioration of complications. e.g., type 2 diabetes, cardiovascular diseases, and hepatocyte carcinoma (Younossi et al., 2018). Although NAFLD often co-exists with obesity, a substantial proportion of NAFLD patients are lean, with a prevalence of 5%–45% in Asians (Eslam et al., 2020) caused by diagnostic strategy selection and genetic variation induced by ethnicity, which is relatively unexplored.

NAFLD may be highly susceptible to genetic and environmental factors (Eslam and George, 2016; Eslam et al., 2018). It was estimated that the heritability of hepatic fat content in NAFLD in the general population was nearly 52 percent (Loomba et al., 2015). Moreover, genetic variations found in the genome-wide association study (GWAS) also govern the development of NAFLD (Dongiovanni et al., 2015; Anstee et al., 2020; Trepo and Valenti, 2020). The single nucleotide polymorphisms (SNPs) strongly related to the accumulation of triglyceride and inflammation in liver disease, i.e., rs738409 C>G polymorphism in patatin‐like phospholipase domain containing 3 (*PNPLA3*), rs58542926 C>T variant in transmembrane 6 superfamily member 2 (*TM6SF2*), and rs641738C>T in membrane-bound O-acyltransferase domain-containing 7 (*MBOAT7*) were the most robust and well-replicated locus (Eslam et al., 2018). Notably, the distribution of the G allele in *PNPLA3* and the T allele of *TM6SF2* was much higher in lean NAFLD than in non-lean NAFLD (Feldman et al., 2017; Chen et al., 2020). Except the above SNPs involved in the progression of NAFLD or lean NAFLD, other potential candidate SNPs’ actions in the treatment of NAFLD or lean NAFLD are also worth investigating, which would provide some clues for the clarification of the pathogenesis of lean NAFLD.

Instead of drugs, physical exercise and energy intake control are prioritized for the treatment of NAFLD. According to the finding, lean NAFLD patients had high fructose, and cholesterol intake (Wong et al., 2018), and the genes strongly linked with glucose or lipid metabolism captured much attention [e.g., insulin receptor substrate 1 (*IRS1*) and leptin (*LEP*)]. As an energy-sensing enzyme, AMP-activated protein kinase (AMPK) seems to perform a vital role in preventing NAFLD (Smith et al., 2016). The activation of the AMPK a2 subunit in the liver can decrease blood glucose levels and inhibit gluconeogenesis (Foretz et al., 2005). Moreover, the deletion of AMPK can reduce the thermogenesis of brown adipose tissue and exacerbate insulin resistance and hepatic steatosis (Desjardins and Steinberg, 2018). Yet the role of the genetic variant in genes clustered in AMPK signaling pathway of lean NAFLD was still unclear.

In this study, we focused on the NAFLD-related genes [e.g., TBC1 domain family member 1, (*TBC1D1*)] enriched in the AMPK signaling pathway and aimed to examine the association between sixteen common polymorphisms and the risk of lean NAFLD in the elderly Chinese Han population. This knowledge may help identify the genetic variations contributing to the development of lean NAFLD and imply the strategy for the prevention and control of lean NAFLD.

## Materials and methods

### Subjects

Data were collected in a cross-sectional survey among 5387 residents (aged ≥60 years) in the Zhangjiang community center, Shanghai, China, in 2017. The study was approved by the Ethics Committee of Shanghai University of Traditional Chinese Medicine. Written informed consent was provided before the study. The study followed the declaration of Helsinki.

**FIGURE 1:**
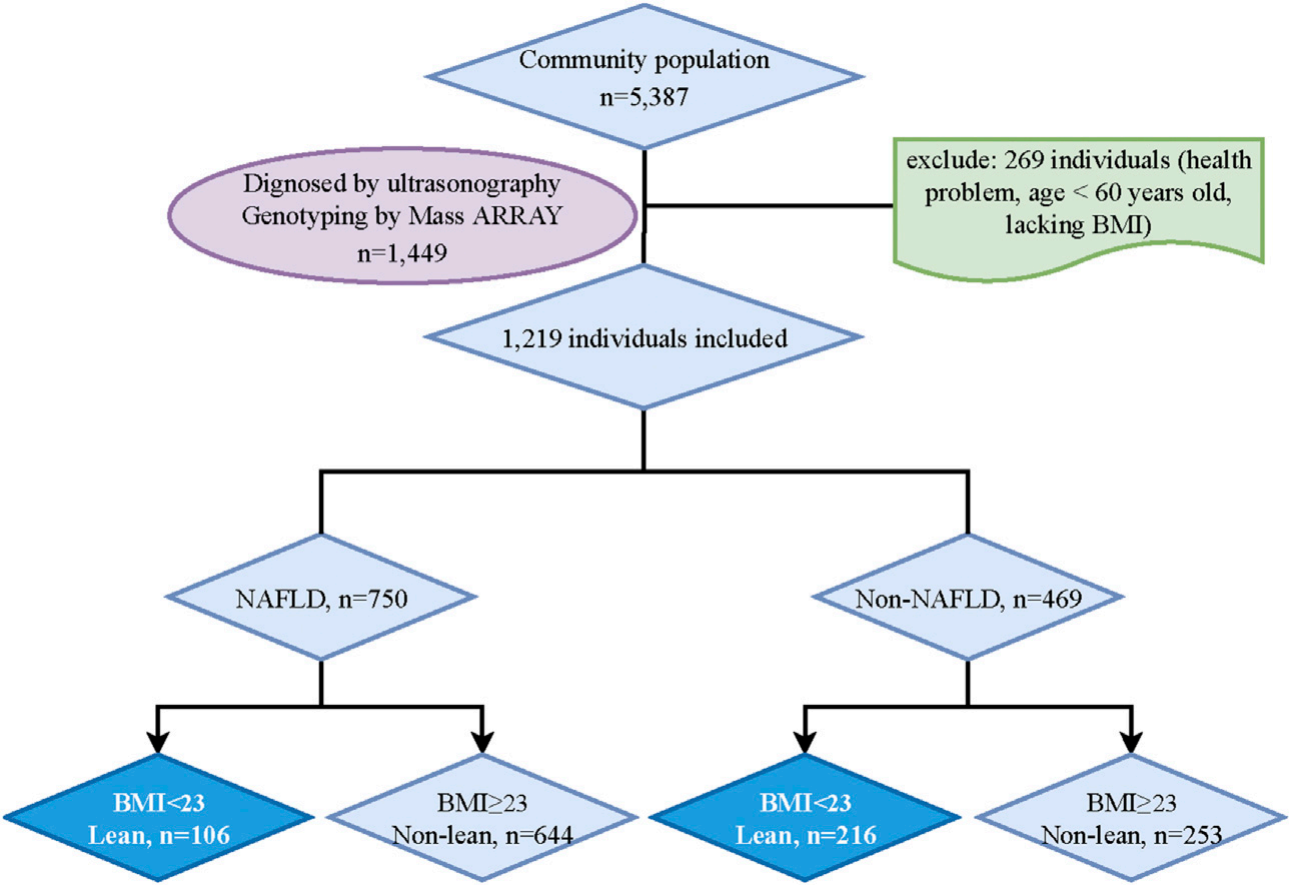
Study flowchart. BMI, Body mass index; NAFLD, Non-alcoholic fatty liver disease.

The residents in Shanghai who can complete the data measurements were included in this study. And participants with mental disorders, malignant tumors, or incomplete recorded information would be excluded. After an initial screening, 5338 potential subjects were included. Then, 1449 participants were randomly chosen for the genotyping analysis. However, 230 participants who lacked BMI data, abused alcohol (<140 g/week in males and <70 g/week in females), were carriers of hepatitis B or C, or had a history of drug-induced liver disease or autoimmune liver disease were excluded. Ultimately, 1219 participants (NAFLD, *n* = 750; non-NAFLD, *n* = 469) were included in the final analysis. According to the classification in adult Asian populations, lean NAFLD in this study was defined by body mass index (BMI) < 23 kg/m^2^ (Fan et al., 2017; Vilarinho et al., 2021). Finally, participants were categorized into four groups: lean NAFLD (BMI <23 kg/m^2^, *n* = 106), non-lean NAFLD (BMI ≥23 kg/m^2^, *n* = 644), lean non-NAFLD (BMI <23 kg/m^2^, *n* = 216) and non-lean non-NAFLD (BMI ≥23 kg/m^2^, *n* = 253) (Figure 1).

### Measurement

A color ultrasound system was used to diagnose NAFLD (Fan et al., 2011) by two experienced radiologists, and it can be defined by the presence of at least two of three abnormal findings: diffusely increased liver near field ultrasound echo (‘bright liver’), liver echo greater than kidney, vascular blurring and the gradual attenuation of far field ultrasound echo. Information, i.e., age, gender, alcohol consumption, smoking, and medical history, was acquired by questionnaire. BMI was calculated as weight (kg) divided by height squared (m^2^). A non-stretch tape was used for the measurement of waist and hip circumference (WC/HC) by the trained professional. Electronic sphygmomanometers (Biospace, Cheonan, South Korea) were used to measure diastolic and systolic blood pressure (DBP and SBP). A biochemistry analyzer (Hitachi, Tokyo, Japan) was applied for testing fasting glucose, alanine transaminase (ALT), aspartate transaminase (AST), total cholesterol (TC), low-density lipoprotein (LDL), high-density lipoprotein (HDL) and triglyceride (TG).

### Kyoto encyclopedia of genes and genomes (KEGG) pathway analysis

KEGG pathway analysis was performed in the online software DAVID (Sherman et al., 2022) and a *p*-value less than .05 was considered statistically significant. 116 NAFLD-related candidate genes, which cover 179 SNPs (Supplementary Table S1) were included in the preliminary selection for the enrichment analysis. Due to the vital roles of the AMPK signaling pathway in NAFLD (Smith et al., 2016), only seven genes [*TBC1D1*, *IRS1*, *LEP*, adiponectin, C1Q and collagen domain-containing (*ADIPOQ*), leptin receptor (*LEPR*), peroxisome proliferator-activated receptor gamma (*PPARG*) and sirtuin (*SIRT1*)] involved in the AMPK signaling pathway were described in detail in the following genotyping section to clarify the biological function of genetic variation occurring in the specific genes of lean NAFLD.

### Genotyping

The genomic DNA was extracted from peripheral venous blood using the standard phenol-chloroform method for genotyping by collecting 5 mL of blood from each subject. Sixteen SNPs including: rs2279026 and rs72279028 in *TBC1D1*, rs1801278 in *IRS1*, rs3828942 in *LEP*, rs182052, rs6773957, rs3774261 and rs17366568 in *ADIPOQ*, rs11208659, rs12409877, rs1805094 and rs4655537 in *LEPR*, rs1801282 in *PPARG*, rs11599176, rs12413112 and rs33957861 in *SIRT1* from NCBI database of SNP database were analyzed. Matrix-assisted laser desorption/ionization time-off light mass spectrometer in MassARRAY Analyzer 4 platforms (Sequenom, San Diego, CA) was used for genotyping. Probes and primers were designed with online Assay Design Suite version 2.0 software. The polymerase chain reaction was performed according to the instructions of the manufacturers. More detailed information about primers and polymerase chain reaction conditions is available upon request.

### Statistical analysis

Basic traits in participants were presented as mean and standard deviation (SD). Independent samples *t*-test was adopted for the group comparison. Categorical data were calculated as a percentage. Non-normally distributed data were analyzed by converting log to normally distributed data, and non-parametric testing, i.e., Mann-Whitney U, was used for data that cannot be converted into normally distributed data. Allelic and genotypic distributions and Hardy-Weinberg equilibrium were analyzed with the online software SHEsis (http://analysis.bio-x.cn/myAnalysis.php) (Shi and He, 2005).

“SNPassoc” R package (Gonzalez et al., 2007) was applied for the association analysis between SNPs with phenotypes in five genetic models (codominant, dominant, recessive, over-dominant, and log-additive models, respectively). To verify the association of HDL with rs2279028 or ALT/LDL/TC with rs17366568 in lean NAFLD, the logistic regression analysis was used. Only those statistically significant variables in both genetic association and regression analysis will be included in the subsequent mediation analysis. Mediation models conducted with mediation package in R software were used to explore whether specific phenotypes can mediate the association between SNPs and the risk of lean NAFLD. A recommended procedure with 95% confidence intervals of 1,000 bias-corrected and accelerated bootstrapping analyses were used to assess the significance of mediation effects. *p* < .05 was considered significant in this study.

## Results

### Demographics of study participants

**FIGURE 2:**
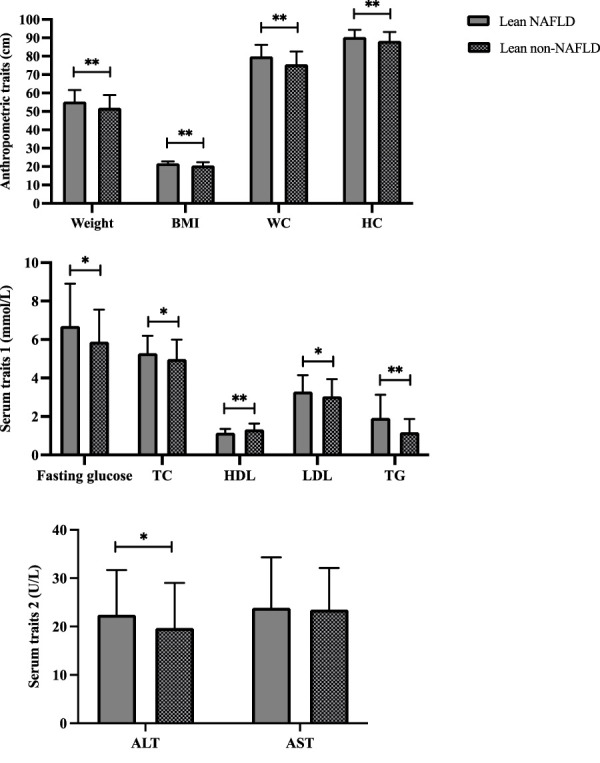
Demographics of lean NAFLD and lean non-NAFLD participants. ALT, Alanine aminotransferase; AST, Aspartate aminotransferase; BMI, Body mass index; HDL, High-density lipoprotein; LDL, Low-density lipoprotein; NAFLD, Non-alcoholic fatty liver disease; TC, Total cholesterol; TG, Triglyceride. * and ** indicate p < 0.05 and p < 0.001, respectively.

Demographics of the lean participants are presented in Table 1 and Figure 2. Females constituted 64.2 and 58.8 percent of lean NAFLD (average age: 72.5 years) and lean non-NAFLD participants (average age: 73.5 years), respectively. And males accounted for 35.8 and 41.2 percent of lean NAFLD and lean non-NAFLD participants, respectively. In lean NAFLD patients, the weight, BMI, WC, and HC level was much higher than in lean non-NAFLD individuals (*p* < .05). And the serum trait, i.e., fasting glucose, TC, LDL, TG, and ALT, were statistically greater in lean NAFLD compared with lean non-NAFLD individuals (*p* < .05). By contrast, HDL was much lower in lean NAFLD than in lean non-NAFLD individuals (*p* < .05).

**TABLE 1:** Demographics of lean NAFLD and lean non-NAFLD participants.

	Lean NAFLD	Lean non-NAFLD	*p*-value
	Mean ± SD	Mean ± SD	
N	106	216	.169
Age (year)	72.54 ± 6.05	73.54 ± 6.15	
Gender (%)			.357
Female	64.20%	58.80%	
Male	35.80%	41.20%	
SBP (mmHg)	142.04 ± 20.43	138.04 ± 22.45	.124
DBP (mmHg)	81.08 ± 12.32	78.83 ± 11.92	.119
Hypertension (%)	55.70	44.00	.050
T2D (%)	20.80	12.00	.056
Hyperlipidemia (%)	10.40	6.500	.221

Variables are presented as mean ± SD. *p* values are based on independent sample *t*-test. DBP, diastolic blood pressure; NAFLD, Non-alcoholic fatty liver disease; SBP, systolic blood pressure; SD, standard deviation; T2D, Type 2 diabetes.

### KEGG enrichment analysis based on the candidate SNPs related to NAFLD

According to KEGG pathway analysis, the candidate genes (SNPs) were significantly enriched in eight pathways related to NAFLD (*p* < .05) (Table 2). Among them, the AMPK signaling pathway, which includes *TBC1D1*, *IRS1*, *LEP*, *ADIPOQ*, *LEPR*, *PPARG*, and *SIRT1* genes, was the most significant (*p* < .001). As the AMPK signaling pathway plays an essential role in the development of NAFLD (Smith et al., 2016; Carling, 2017), we next focused on the sixteen SNPs in the seven genes mentioned above.

**TABLE 2:** KEGG pathway enrichment analysis of the candidate genes related to NAFLD.

Term	*p*-value	FDR	Genes
AMPK signaling pathway	<.001	.066	TBC1D1, IRS1, LEP, ADIPOQ, LEPR, PPARG, SIRT1
Cholesterol metabolism	.001	.066	CETP, NPC1, LPL, APOC3, APOE
PPAR signaling pathway	.003	.198	FADS2, ADIPOQ, LPL, APOC3, PPARG
Longevity regulating pathway	.006	.275	IRS1, ADIPOQ, PPARG, SOD2, SIRT1
Non-alcoholic fatty liver disease	.008	.316	IRS1, LEP, ADIPOQ, LEPR, PPARG, IL6R
Adipocytokine signaling pathway	.018	.580	IRS1, LEP, ADIPOQ, LEPR
Alcoholic liver disease	.028	.759	TCF7L2, ADIPOQ, LPIN1, SIRT1, TLR4
Nicotinate and nicotinamide metabolism	.033	.771	ENPP1, NT5C2, SIRT1
Type 2 diabetes	.054	1.000	KCNJ11, IRS1, ADIPOQ
Neurotrophin signaling pathway	.072	1.000	IRS1, BDNF, SH2B3, SH2B1
Glycerolipid metabolism	.088	1.000	PNPLA3, LPL, LPIN1

AMPK, AMP-activated protein kinase; PPAR, peroxisome proliferator-activated receptors.

### Genetic association between SNPs and lean NAFLD

Sixteen tested SNPs (rs2279026 and rs72279028) in *TBC1D1*, (rs1801278) in *IRS1*, (rs3828942) in *LEP*, (rs182052, rs6773957, rs3774261 and rs17366568) in *ADIPOQ*, (rs11208659, rs12409877, rs1805094 and rs4655537) in *LEPR*, (rs1801282) in *PPARG*, (rs11599176, rs12413112 and rs33957861) in *SIRT1* which met Hardy-Weinberg equilibrium (*p* > .05) were shown in Table 3. The frequency of above sixteen SNPs between males and females is shown in supplementary Table 2.

**TABLE 3:** The sixteen SNPs analyzed in this study.

Gene	SNP Id	Chromosome	Function	Allele
TBC1D1	rs2279027	Chr4:37902135	missense_variant	T/A
	rs2279028	Chr4:37902048	genic_upstream_transcript_variant	A/G
IRS1	rs1801278	Chr2:226795828	missense_variant	C/A
LEP	rs3828942	Chr7:128254252	intron_variant	G/A
ADIPOQ	rs182052	Chr3:186842993	intron_variant	G/A
	rs6773957	Chr3:186855916	non_coding_transcript_variant	A/C
	rs3774261	Chr3:186853770	intron_variant	A/G
	rs17366568	Chr3:186852664	non_coding_transcript_variant	G/A
LEPR	rs11208659	Chr1:65513597	genic_upstream_transcript_variant	T/A
	rs12409877	Chr1:65478189	intron_variant	A/G
	rs1805094	Chr1:65610269	missense_variant	G/C
	rs4655537	Chr1:65593118	intron_variant	A/G
PPARG	rs1801282	Chr3:12351626	coding_sequence_variant	C/G
SIRT1	rs11599176	Chr10:67894017	intron_variant	A/G
	rs12413112	Chr10:67892108	intron_variant	G/A
	rs33957861	Chr10:67887218	intron_variant	C/T

ADIPOQ, adiponectin, C1Q and collagen domain containing; Chr, Chromosome; IRS1, Insulin receptor substrate 1; LEP, leptin; LEPR, leptin receptor; OR, odds ratio; PPARG, Peroxisome proliferator-activated receptor gamma; SIRT1, Sirtuin 1; SNP, single nucleotide polymorphisms; TBC1D1, TBC1 domain family member 1.

The allele and genotype distributions of sixteen SNPs are shown in Table 4. The A allele frequency of rs2279028 in *TBC1D1* was significantly lower in lean NAFLD comparing with lean non-NAFLD individuals (OR = .603, 95%CI = .419–.866, *p* = .006, adjusted *p* = .046). The genotypic frequency of rs2279028 was significantly different between lean NAFLD and lean non-NAFLD individuals (*p* = .024). Moreover, G allele frequency of rs17366568 in *ADIPOQ* was lower in lean NAFLD comparing with lean non-NAFLD individuals (OR = 2.625, 95%CI = 1.020–6.752, *p* = .038). However, there were no significant differences between the allele or among the genotype distributions of the other fourteen SNPs (Supplementary Table 3).

**TABLE 4:** Analyzed genes allele and genotype distribution in lean individuals (Cont. see Additional file 1).

Gene	SNP	Allele Frequency		χ2	P	FDR adjusted	OR (95% CI)	Genotype Frequency			χ2	P	FDR adjusted	HWE
TBC1D1	rs2279027	C	T	1.242	.264	.593	1.212 (.863–1.701)	C/C	T/C	T/T	1.613	.446	.637	
	Lean NALFD	128 (.603)	84 (.396)					38 (.358)	52 (.49)	16 (.15)				.966
	Lean non-NAFLD	279 (.648)	151 (.351)					93 (.432)	93 (.432)	29 (.134)				.757
	rs2279028	G	A	7.526	.006	.046	.603 (.419–.866)	G/G	A/G	A/A	7.396	.024	.156	
	Lean NALFD	56 (.266)	154 (.733)					8 (.076)	40 (.380)	57 (.542)				.965
	Lean non-NAFLD	161 (.376)	267 (.623)					31 (.144)	99 (.462)	84 (.392)				.978

FDR, false discovery rate; ADIPOQ, adiponectin, C1Q and collagen domain containing; Cont., continued; HWE, Hardy-Weinberg equilibrium; IRS1, Insulin receptor substrate 1; LEP, leptin; LEPR, leptin receptor; OR, odds ratio; PPARG, Peroxisome proliferator-activated receptor gamma; SIRT1, Sirtuin 1; SNP, single nucleotide polymorphisms; TBC1D1, TBC1 domain family member 1.

### Association of SNPs with specific phenotypes in lean NAFLD individuals

Only associations between two SNPs, i.e., rs2279028 in *TBC1D1* and rs17366568 in *ADIPOQ,* and specific phenotypes, e.g., HDL, ALT, LDL, and TC, were significant. The association between the polymorphism in *TBC1D1* and HDL using five genetic models is presented in Table 5. *TBC1D1* polymorphism rs2279028 was significantly associated with HDL under the over-dominant model. The GA genotype of rs2279028 polymorphism was statistically related to lower HDL (*p* = .035). We also used rs2279028 as a logistic regression predictor to examine its associations with HDL. And rs2279028 genotype was still significant associated with HDL (*β* = -.084, *R* ^2^ = .054, *p* = .009) after adjusting gender and age (Table 7).

**TABLE 5:** Association between *TBC1D1* SNPs and HDL.

Association	Genotype	N	95%CI	*p*-value
rs2279028-HDL	Codominant			
	A/A	138		.104
	G/A	137	−.003–.132	
	G/G	39	−.118–.085	
	Dominant			
	A/A	138		.152
	G/A-G/G	176	−.017–.110	
	Recessive			
	A/A-G/A	275		.317
	G/G	39	−.145–.047	
	Over-dominant			
	A/A-G/G	177		.035
	G/A	137	.005–.132	
	log-Additive			
	0,1,2		−.033–.577	.060

CI, confidence interval; HDL, High-density lipoprotein; SNP, single nucleotide polymorphisms; TBC1D1, TBC1 domain family member 1.

The association between *ADIPOQ* SNPs and specific phenotypes using five genetic models are presented in Table 6. *ADIPOQ* polymorphism rs17366568 was significantly associated with ALT under the codominant, dominant and over-dominant models (*p* = .024, *p* = .023 and *p* = .010, respectively). Moreover, rs17366568 was significantly associated with LDL under the codominant and recessive models (*p* = .041 and *p* = .014, respectively). In addition, rs17366568 was significantly associated with TC under the recessive model (*p* = .032). Similarly, rs17366568 was also used as a predictor of logistic regression to examine its associations with ALT, LDL, and TC, respectively. And all the associations were significant (rs17366568 vs. ALT: *β* = -5.083, *R* ^2^ = .082, *p* = .028; rs17366568 vs. LDL: *β* = -2.056, *R* ^2^ = .066, *p* = .018; rs17366568 vs. TC: *β* = -2.023, *R* ^2^ = .140, *p* = .029) after adjusting gender and age (Table 7).

**TABLE 6:** Association between *ADIPOQ* SNPs and specific phenotypes.

Association	Genotype	N	95%CI	*p*-value
rs17366568-ALT	Codominant			
	G/G	297		.024
	G/A	16	1.477–10.745	
	A/A	1	−26.413–9.759	
	Dominant			
	G/G	297		.023
	G/A-A/A	17	.750–9.774	
	Recessive			
	G/G-G/A	313		.354
	A/A	1	−26.888–9.609	
	Over-dominant			
	G/G-A/A	298		.010
	G/A	16	1.507–10.771	
	log-Additive			
	0,1,2		−.159–8.172	.060
rs17366568-LDL	Codominant			
	G/G	297		.041
	G/A	16	−.572–.310	
	A/A	1	.444–3.886	
	Dominant			
	G/G	297		.986
	G/A-A/A	17	−.428–.436	
	Recessive			
	G/G-G/A	313		.014
	A/A	1	.459–3.891	
	Over-dominant			
	G/G-A/A	298		.542
	G/A	16	−.583–.306	
	log-Additive			
	0,1,2		−.280–.515	.563
rs17366568-TC	Codominant			
	G/G	297		.093
	G/A	16	−.590–.395	
	A/A	1	.186–4.030	
	Dominant			
	G/G	297		.896
	G/A-A/A	17	−.449–.514	
	Recessive			
	G/G-G/A	313		.032
	A/A	1	.193–4.032	
	Over-dominant			
	G/G-A/A	298		.679
	G/A	16	−.600–.391	
	log-Additive			
	0,1,2		−.305–.581	.541

ADIPOQ, adiponectin, C1Q and collagen domain containing; ALT, alanine aminotransferase; CI, confidence interval; LDL, Low-density lipoprotein; SNP, single nucleotide polymorphisms; TC, total cholesterol.

**TABLE 7:** Regression analysis on the impacts of candidate genes’ SNPs on specific phenotypes.

Gene	SNP	Phenotype	Statistics of regression analyses			
			β	R^2^	t	p
TBC1D1	rs2279028	HDL	−.084	.054	−2.635	.009
ADIPOQ	rs17366568	ALT	−5.083	.082	−2.205	.028
	rs17366568	LDL	−2.056	.066	−2.385	.018
	rs17366568	TC	−2.023	.140	−2.193	.029

ADIPOQ, adiponectin, C1Q and collagen domain containing; SNP, single nucleotide polymorphisms; TBC1D1, TBC1 domain family member 1.

### Mediation effect of the specific phenotype on the association between SNPs and the risk of lean NAFLD

**FIGURE 3:**
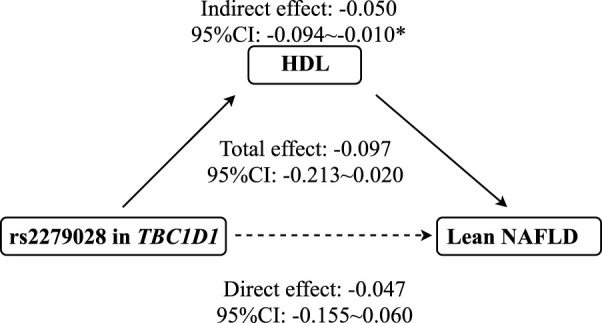
The mediation effect of HDL on the association between rs2279028 and the risk of lean NAFLD. CI, Confidence interval; HDL, High-density lipoprotein; NAFLD, Non-alcoholic fatty liver disease; TBC1D1, TBC1 domain family member 1. * indicates p < 0.05.

As shown in Figure 3, mediation analysis showed that rs2279026 had no significant direct effect on lean NAFLD (*β* = -.047, 95%CI: -.155–.060), while rs1260326 had a significant indirect effect on lean NAFLD incidence *via* HDL (*β* = -.050, 95%CI: -.094∼-.010, *p* = .014). In addition, ALT, LDL, and TC have no mediation effects on the association between 17366568 in ADIPOQ and the risk of lean NAFLD.

## Discussion

There were significant differences in anthropometric measures (e.g., weight, BMI, WC, and HC), serum parameters (e.g., TC, LDL, HDL, and TG), and allele and genotype distributions (e.g., rs2279028 in *TBC1D1*) between lean NAFLD and lean non-NAFLD participants. Moreover, polymorphisms in *TBC1D1* and *ADIPOQ,* which are involved in the AMPK signaling pathway, were linked with cardiovascular risk factors (i.e., HDL, LDL, TC, and TG). This study reinforced the mediation effect of HDL on the association between rs2279028 in *TBC1D1* and the risk of lean NAFLD in the elderly Chinese Han population for the first time.

Currently, the pathophysiology of lean NAFLD remains to be resolved incompletely. The impaired glucose tolerance with increased hepatic fat accumulation and low adiponectin concentrations has been proved in obese and lean NAFLD patients (Feldman et al., 2017). It was reported that not only does insulin resistance contribute to obese NAFLD, but lean NAFLD (Bugianesi et al., 2005). Furthermore, insulin resistance was associated with reduced HDL (Fossati and Romon-Rousseaux, 1987). Notably, the antiatherogenic factor, i.e., HDL, could activate AMPK, which is highly sensitive to glucose (Lin and Hardie, 2018). All these findings hint at the crucial role of HDL in lean NAFLD. Additionally, in cases with a lack of AMPK in adipocytes, insulin resistance and simple steatosis were triggered and aggravated (Mottillo et al., 2016). And the symptom (i.e., steatosis and inflammation) of NAFLD was improved through the AMPK signaling pathway after exercise intervention in obese mice (Diniz et al., 2021). Thus, we proposed that AMPK might play a vital role in the occurrence, development, prevention, and even treatment of lean NAFLD *via* regulating glucose and lipid metabolism.

Lean NAFLD might result from genetic variation in genes related to AMPK signaling due to its regulatory effect on glucose and lipid. *TBC1D1* is the downstream target of AMPK, and glucose uptake by AMPK in skeletal muscle needs the phosphorylation of *TBC1D1* (Thomas et al., 2018). And *ADIPOQ* deficiency might lead to insulin resistance and NAFLD by mediating the AMPK signaling pathway (Shehzad et al., 2012). But more than that, different SNPs in NAFLD-related genes, e.g., *PNPLA3*, *ADIPOQ*, and *TBC1D1,* appear to significantly influence the development of lean NAFLD. Zou et al. found that the number of lean NAFLD patients carrying rs738409 polymorphism in *PNPLA3* was much greater than obese NAFLD and lean controls (Zou et al., 2020). In addition, Hsiao et al. reported that rs266729 in *ADIPOQ* was associated with TC in obese subjects but not in non-obese ones (Hsiao and Lin, 2016). Of note, Apalasamy et al. confirmed that rs17366568 in *ADIPOQ* was related to the risk of obesity (Apalasamy et al., 2014). Although the association between polymorphism in *ADIPOQ* and the risk of lean NAFLD failed to fit the mediation model, our study still showed that rs17366568 was significantly correlated with TC, LDL, and TG after the regression analysis, which is in agreement with the previous findings. Researchers have implied that *TBC1D1* could determine fat and muscle deposition. Knuppel et al. demonstrated that rs637797 in *TBC1D1* was strongly linked with BMI (Knuppel et al., 2013). And Fontanesi et al. explored the association between the genetic variant in *TBC1D1* and fat thickness and lean mass (Fontanesi et al., 2011). Chadt et al. showed that mutation of *TBC1D1* could inhibit obesity by promoting the utilization of lipids (Chadt et al., 2008). Consistently, our study showed that rs1260328 polymorphism in *TBC1D1* was associated with the risk of lean NAFLD *via* HDL, which further partially supported the above assumption.

The advantage of this study is that it is the first time to examine the mediation effect of HDL on the association between rs2279028 and the risk of lean NAFLD in the elderly Chinese Han population. And this research on further pathogenesis mechanism of lean NAFLD has great significance. The limitation of this study is that the mediation effect of HDL on the association between rs2279028 and the risk of lean NAFLD was acquired only based on the elderly Chinese Han population; more similar studies should be conducted to validate the finding in this manuscript which should cover different populations with more sample sizes, diverse age, ethics, regions and environmental exposure factors for avoiding the potential confounding factors. In addition, GGT, as a key marker of liver injury, should also be tested for reflecting hepatocellular damage.

## Conclusion

In conclusion, this study indicated that rs2279028 in *TBC1D1* may contribute to the progression of lean NAFLD through HDL. This finding presents more evidence for further exploring the mechanism of lean NAFLD, and suggests the possible strategy for the prevention and treatment of lean NAFLD.

## Data Availability

The datasets presented in this study can be found in online repositories. The names of the repository/repositories and accession number(s) can be found below: https://pan.baidu.com/s/1LgXoUgWFZ162l8zE8cyv_w, 1rc3.

## Supplementary material

SUPPLEMENTARY FIGURE S1 Mediation effect of variables on the association between rs17366568 in ADIPOQ and the risk of lean NAFLD.

SUPPLEMENTARY TABLE S1 179 SNPs included in this study (see the online repository).

SUPPLEMENTARY TABLE S2 The frequency of above sixteen SNPs between males and females.

SUPPLEMENTARY TABLE S3 Cont. Analyzed genes allele and genotype distribution in lean individuals.
