# Supplementary material for: Genetic variation in TBC1 domain family member 1 gene associates with the risk of lean NAFLD via high-density lipoprotein
Source: Front Genet. 2023 Jan 12;13:1026725. doi: 10.3389/fgene.2022.1026725 (PMC9877292; doi:10.3389/fgene.2022.1026725)
Supplement: Supplementary file 1 [file Table2.docx]

**Supplementary Table S2 The frequency of above sixteen SNPs between males and females**

|  |  | **Lean NAFLD** | | | **Lean non-NAFLD** | | |
| --- | --- | --- | --- | --- | --- | --- | --- |
|  |  | **Male (%)** | **Female (%)** | **P-value** | **Male (%)** | **Female (%)** | **P-value** |
| rs2279027 | C C | 43.80 | 42.50 | 0.571 | 34.20 | 36.80 | 0.515 |
|  | C T | 43.80 | 42.50 |  | 55.30 | 45.60 |  |
|  | T T | 11.20 | 15.00 |  | 10.50 | 17.60 |  |
| rs2279028 | A A | 30.30 | 44.90 | 0.195 | 57.90 | 51.50 | 0.442 |
|  | G A | 51.70 | 41.70 |  | 39.50 | 36.80 |  |
|  | G G | 16.90 | 12.60 |  | 2.60 | 10.30 |  |
| rs1801278 | C C | 93.30 | 97.60 | 0.101 | 94.70 | 91.20 | 0.745 |
|  | C T | 1.10 | 1.60 |  | 2.60 | 5.90 |  |
| rs3828942 | A A | 55.10 | 50.40 | 0.141 | 47.40 | 54.40 | 0.635 |
|  | G A | 33.70 | 44.90 |  | 44.70 | 33.80 |  |
|  | G G | 10.10 | 4.70 |  | 7.90 | 10.30 |  |
| rs182052 | A A | 19.10 | 17.30 | 0.027 | 10.50 | 22.10 | 0.240 |
|  | A G | 41.60 | 54.30 |  | 60.50 | 41.20 |  |
|  | G G | 31.50 | 27.60 |  | 26.30 | 32.40 |  |
| rs6773957 | A A | 33.70 | 32.30 | 0.658 | 36.80 | 27.90 | 0.551 |
|  | G A | 41.60 | 44.90 |  | 44.70 | 42.60 |  |
|  | G G | 23.60 | 22.80 |  | 18.40 | 27.90 |  |
| rs3774261 | A A | 32.60 | 32.30 | 0.974 | 36.80 | 27.90 | 0.604 |
|  | A G | 41.60 | 44.10 |  | 44.70 | 44.10 |  |
|  | G G | 24.70 | 22.80 |  | 18.40 | 26.50 |  |
| rs17366568 | A A | 0.00 | 0.00 | 0.846 | 0.00 | 1.50 | 0.650 |
|  | G A | 4.50 | 3.10 |  | 5.30 | 8.80 |  |
|  | G G | 94.40 | 96.10 |  | 94.70 | 88.20 |  |
| rs11208659 | T C | 9.00 | 6.30 | 0.364 | 7.90 | 5.90 | 0.689 |
|  | TT | 89.90 | 93.70 |  | 92.10 | 94.10 |  |
| rs12409877 | A A | 76.40 | 81.90 | 0.085 | 78.90 | 88.20 | 0.327 |
|  | A G | 16.90 | 16.50 |  | 18.40 | 7.40 |  |
|  | G G | 0.00 | 0.80 |  | 0.00 | 1.50 |  |
| rs1805094 | G C | 5.60 | 4.70 | 0.465 | 7.90 | 10.30 | 0.685 |
|  | G G | 93.30 | 95.30 |  | 92.10 | 89.70 |  |
| rs4655537 | A A | 2.20 | 1.60 | 0.170 | 0.00 | 0.00 | 0.716 |
|  | G A | 10.10 | 13.40 |  | 18.40 | 14.70 |  |
|  | G G | 82.00 | 84.30 |  | 76.30 | 82.40 |  |
| rs1801282 | C C | 87.60 | 89.00 | 0.682 | 92.10 | 92.60 | 0.701 |
|  | C G | 10.10 | 10.20 |  | 7.90 | 5.90 |  |
|  | G G | 1.10 | 0.80 |  | 0.00 | 0.00 |  |
| rs11599176 | A A | 79.80 | 73.20 | 0.388 | 84.20 | 67.60 | 0.095 |
|  | A G | 18.00 | 25.20 |  | 13.20 | 30.90 |  |
|  | G G | 1.10 | 1.60 |  | 2.60 | 0.00 |  |
| rs12413112 | A A | 1.10 | 1.60 | 0.388 | 2.60 | 0.00 | 0.095 |
|  | A G | 18.00 | 25.20 |  | 13.20 | 30.90 |  |
|  | G G | 79.80 | 73.20 |  | 84.20 | 67.60 |  |
| rs33957861 | C C | 80.90 | 73.20 | 0.537 | 84.20 | 69.10 | 0.059 |
|  | T C | 18.00 | 24.40 |  | 13.20 | 30.90 |  |
|  | T T | 1.10 | 1.60 |  | 2.60 | 0.00 |  |
