# Supplementary material for: Genetic variation in TBC1 domain family member 1 gene associates with the risk of lean NAFLD via high-density lipoprotein
Source: Front Genet. 2023 Jan 12;13:1026725. doi: 10.3389/fgene.2022.1026725 (PMC9877292; doi:10.3389/fgene.2022.1026725)
Supplement: Supplementary file 2 [file Table3.docx]

**SUPPLEMENTARY TABLE 3** Cont. Analyzed genes allele and genotype distribution in lean individuals

| **Gene** | | **SNP** | **Allele Frequency** | | | **χ2** | **P** | **FDR Adjusted** | | **OR (95% CI)** | **Genotype Frequency** | | | **χ2** | **P** | **FDR Adjusted** | **HWE** |
| --- | --- | --- | --- | --- | --- | --- | --- | --- | --- | --- | --- | --- | --- | --- | --- | --- | --- |
| **IRS1** | **rs1801278** | | | C | T | 3.214 | 0.073 | 0.365 | 3.457(0.818-14.61) | | C/C | T/C |  | 3.256 | 0.071 | 0.402 |  |
|  | Lean NALFD | | | 201(0.975) | 5(0.024) |  |  |  |  | | 98(0.951) | 5(0.048) |  |  |  |  | 0.955 |
|  | Lean non-NAFLD | | | 417(0.992) | 3(0.007) |  |  |  |  | | 207(0.985) | 3(0.014) |  |  |  |  | 0.990 |
| **LEP** | **rs3828942** | | | A | G | 0.130 | 0.717 | 0.857 | 1.070(0.741-1.544) | | A/A | G/A | G/G | 0.686 | 0.709 | 0.818 |  |
|  | Lean NALFD | | | 150(0.714) | 60(0.285) |  |  |  |  | | 55(0.523) | 40(0.38) | 10(0.095) |  |  |  |  |
|  | Lean non-NAFLD | | | 313(0.727) | 117(0.272) |  |  |  |  | | 113(0.525) | 87(0.404) | 15(0.069) |  |  |  |  |
| **ADIPOQ** | **rs182052** | | | A | G | 0.020 | 0.886 | 0.950 | 0.975(0.696-1.367) | | A/A | G/G | G/A | 0.039 | 0.980 | 0.998 |  |
|  | Lean NALFD | | | 89(0.436) | 115(0.563) |  |  |  |  | | 19(0.186) | 32(0.313) | 51(0.5) |  |  |  | 0.986 |
|  | Lean non-NAFLD | | | 184(0.442) | 232(0.557) |  |  |  |  | | 39(0.187) | 63(0.302) | 106(0.509) |  |  |  | 0.893 |
|  | **rs6773957** | | | G | A | 0.136 | 0.711 | 0.857 | 1.064 (0.764-1.482) | | G/A | A/A | G/G | 0.122 | 0.940 | 0.945 |  |
|  | Lean NALFD | | | 98(0.466) | 112(0.533) |  |  |  |  | | 46(0.438) | 33(0.314) | 26(0.247) |  |  |  | 0.470 |
|  | Lean non-NAFLD | | | 194(0.451) | 236(0.548) |  |  |  |  | | 94(0.437) | 71(0.33) | 50(0.232) |  |  |  | 0.228 |
|  | **rs3774261** | | | A | G | 0.022 | 0.880 | 0.949 | 1.025 (0.736-1.428) | | A/G | G/G | A/A | 0.063 | 0.968 | 0.968 |  |
|  | Lean NALFD | | | 113(0.538) | 97(0.461) |  |  |  |  | | 47(0.447) | 25(0.238) | 33(0.314) |  |  |  | 0.594 |
|  | Lean non-NAFLD | | | 233(0.544) | 195(0.455) |  |  |  |  | | 93(0.434) | 51(0.238) | 70(0.327) |  |  |  | 0.193 |
|  | **rs17366568** | | | G | A | 4.299 | 0.038 | 0.162 | 2.625 (1.020-6.752) | | G/G | A/G | A/A | 4.326 | 0.114 | 0.376 |  |
|  | Lean NALFD | | | 200(0.952) | 10(0.047) |  |  |  |  | | 96(0.914) | 8(0.076) | 1(0.009) |  |  |  | 0.260 |
|  | Lean non-NAFLD | | | 420(0.981) | 8(0.018) |  |  |  |  | | 206(0.962) | 8(0.037) | 0(0) |  |  |  | 0.961 |
| **LEPR** | **rs11208659** | | | T | C | 0.072 | 0.788 | 0.815 | 0.883 (0.357-2.181) | | T/T | T/C |  | 0.074 | 0.784 | 0.888 |  |
|  | Lean NALFD | | | 205(0.966) | 7(0.033) |  |  |  |  | | 99(0.933) | 7(0.066) |  |  |  |  | 0.928 |
|  | Lean non-NAFLD | | | 414(0.962) | 16(0.037) |  |  |  |  | | 199(0.925) | 16(0.074) |  |  |  |  | 0.878 |
|  | **rs12409877** | | | A | G | 0.951 | 0.329 | 0.787 | 0.729 (0.385-1.378) | | A/G | G/G | A/A | 1.866 | 0.393 | 0.863 |  |
|  | Lean NALFD | | | 192(0.932) | 14(0.067) |  |  |  |  | | 12(0.116) | 1(0.009) | 90(0.873) |  |  |  | 0.717 |
|  | Lean non-NAFLD | | | 380(0.909) | 38(0.09) |  |  |  |  | | 36(0.172) | 1(0.004) | 172(0.822) |  |  |  | 0.830 |
|  | **rs1805094** | | | G | C | 2.091 | 0.148 | 0.444 | 1.885 (0.787-4.513) | | G/G | C/G |  | 2.164 | 0.141 | 0.529 |  |
|  | Lean NALFD | | | 202(0.952) | 10(0.047) |  |  |  |  | | 96(0.905) | 10(0.094) |  |  |  |  | 0.877 |
|  | Lean non-NAFLD | | | 419(0.974) | 11(0.025) |  |  |  |  | | 204(0.948) | 11(0.051) |  |  |  |  | 0.931 |
|  | **rs4655537** | | | G | A | 0.010 | 0.918 | 0.946 | 1.032 (0.562-1.895) | | G/A | A/A | G/G | 2.903 | 0.234 | 0.709 |  |
|  | Lean NALFD | | | 187(0.916) | 17(0.083) |  |  |  |  | | 17(0.166) | 0(0) | 85(0.833) |  |  |  | 0.656 |
|  | Lean non-NAFLD | | | 386(0.919) | 34(0.080) |  |  |  |  | | 26(0.123) | 4(0.019) | 180(0.857) |  |  |  | 0.051 |
| **PPARG** | **rs1801282** | | | C | G | 2.123 | 0.145 | 0.444 | 0.535 (0.228-1.255) | | C/C | C/G | G/G | 2.124 | 0.345 | 0.610 |  |
|  | Lean NALFD | | | 203(0.966) | 7(0.033) |  |  |  |  | | 98(0.933) | 7(0.066) | 0(0) |  |  |  | 0.939 |
|  | Lean non-NAFLD | | | 404(0.939) | 26(0.060) |  |  |  |  | | 191(0.888) | 22(0.102) | 2(0.009) |  |  |  | 0.345 |
| **SIRT1** | **rs11599176** | | | A | G | 0.075 | 0.782 | 0.815 | 1.071 (0.656-1.747) | | A/A | A/G | G/G | 0.328 | 0.848 | 0.888 |  |
|  | Lean NALFD | | | 182(0.866) | 28(0.133) |  |  |  |  | | 78(0.742) | 26(0.247) | 1(0.009) |  |  |  | 0.765 |
|  | Lean non-NAFLD | | | 376(0.874) | 54(0.125) |  |  |  |  | | 164(0.762) | 48(0.223) | 3(0.013) |  |  |  | 0.970 |
|  | **rs12413112** | | | G | A | 0.075 | 0.782 | 0.815 | 1.071 (0.656-1.747) | | G/G | A/G | A/A | 0.328 | 0.848 | 0.888 |  |
|  | Lean NALFD | | | 182(0.866) | 28(0.133) |  |  |  |  | | 78(0.742) | 26(0.247) | 1(0.009) |  |  |  | 0.765 |
|  | Lean non-NAFLD | | | 376(0.874) | 54(0.125) |  |  |  |  | | 164(0.762) | 48(0.223) | 3(0.013) |  |  |  | 0.970 |
|  | **rs33957861** | | | C | T | 0.100 | 0.751 | 0.865 | 1.082 (0.662-1.768) | | C/C | C/T | T/T | 0.384 | 0.825 | 0.870 |  |
|  | Lean NALFD | | | 184(0.867) | 28(0.132) |  |  |  |  | | 79(0.745) | 26(0.245) | 1(0.009) |  |  |  | 0.771 |
|  | Lean non-NAFLD | | | 377(0.876) | 53(0.123) |  |  |  |  | | 165(0.767) | 47(0.218) | 3(0.013) |  |  |  | 0.985 |

FDR: False discover rate; ADIPOQ: Adiponectin, C1Q and collagen domain containing; Cont.: Continued; IRS1: Insulin receptor substrate 1; LEP: Leptin; LEPR: Leptin receptor; OR: Odds ratio; PPARG: Peroxisome proliferator-activated receptor gamma; SIRT1: Sirtuin 1; HWE: Hardy-Weinberg equilibrium; SNP: Single nucleotide polymorphisms; TBC1D1: TBC1 domain family member 1.
