# Supplementary material for: Genetic variation in TBC1 domain family member 1 gene associates with the risk of lean NAFLD via high-density lipoprotein
Source: Front Genet. 2023 Jan 12;13:1026725. doi: 10.3389/fgene.2022.1026725 (PMC9877292; doi:10.3389/fgene.2022.1026725)

SUPPLEMENTARY FIGURE 1 Mediation effect of variables on the association between rs17366568 in ADIPOQ and the risk of lean NAFLD

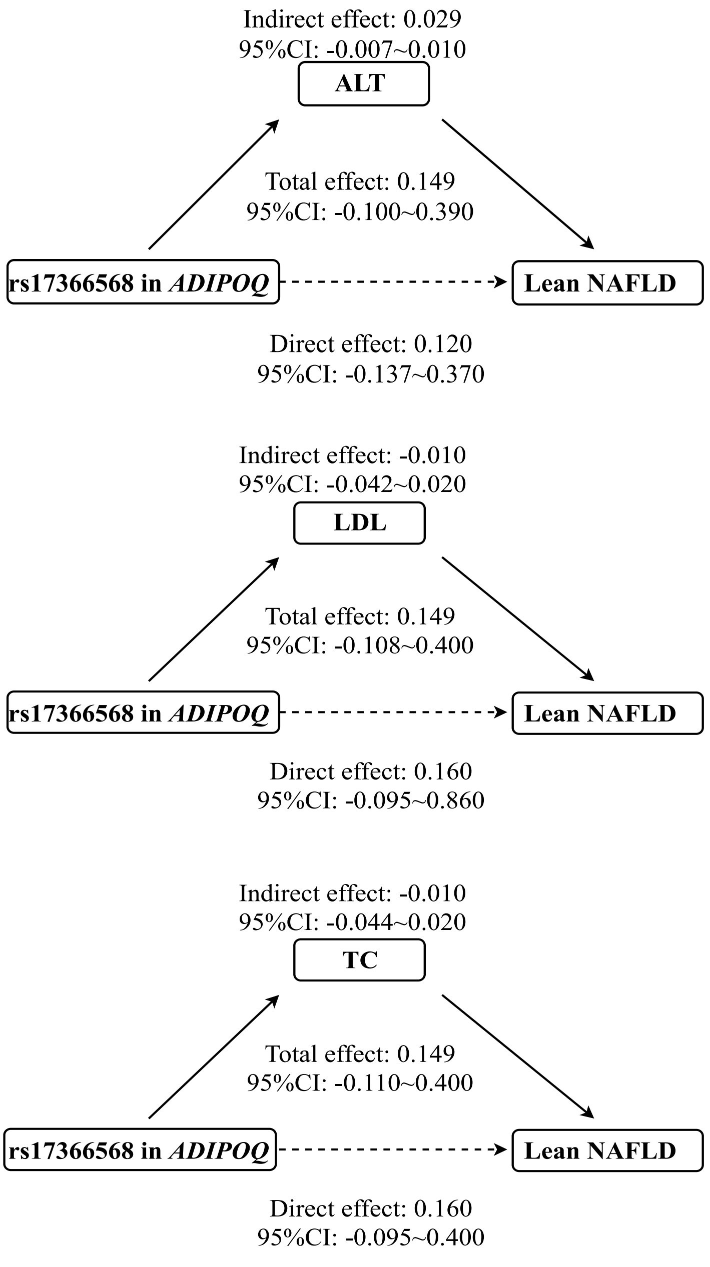

Supplement: Supplementary file 3 [file Image1.pdf]
